# Accidental Left Circumflex Artery to Right Lung Fistula in a Suspected Case of Pulmonary Hypertension

**DOI:** 10.1155/2014/427045

**Published:** 2014-07-17

**Authors:** Saeed Alipourparsa, Isa Khaheshi, Vahid Eslami, Mohammadreza Bozorgmanesh, Habib Haybar

**Affiliations:** ^1^Cardiovascular Research Center, Modarres Hospital, Shahid Beheshti University of Medical Sciences, Tehran, Iran; ^2^Prevention of Metabolic Disorders Research Center, Research Institute for Endocrine Sciences, Shahid Beheshti University of Medical Sciences, Tehran, Iran; ^3^Cardiovascular Research Center, Ahvaz Jundishapur University of Medical Sciences, Ahvaz, Iran

## Abstract

A 56-year-old woman was referred to the cardiology department of the Shahid Modarres hospital. The patient had a history of pulmonary thromboembolism 20 years ago which had been managed by the inferior vena cava filter and since then the patient has been on warfarin. Her chief complaint was chronic dyspnea on exertion (NYHA class II) from several years ago. Right and left heart catheterization was performed for evaluation of pulmonary artery pressure. We found rich collateral formations between LCX as well as RCA and right pulmonary artery, primarily assumed as multiple fistulas. Among patients who have chronic thromboembolic pulmonary hypertension, systemic collateral supply to the pulmonary parenchyma has been previously reported to occur from both bronchial and/or nonbronchial systemic circulations. Our patient had neither signs of heart failure nor myocardial ischemia and, thus, was a candidate for conservative management. The adenosine pulmonary reactivity test was not performed because of low pulmonary pressure which had been estimated to be high.

## 1. Introduction

The case reported herein was a 56-year-old woman with chronic excretion dyspnea (NYHA class II) since 20 years ago when she survived a pulmonary thromboembolism that was managed by the inferior vena cava filter and warfarin. Cardiac examination revealed an irregular rhythm with holosystolic murmur at left lateral sternal border. The physical examination was otherwise unremarkable. On ECG, rhythm was atrial fibrillation (74 bpm) and no significant ST-T changes. Transthoracic echocardiography showed a normal sized LV (LVEDD = 45 mm), no regional wall motion abnormality, moderate right ventricle (RV) enlargement with a right ventricular end diastolic dimension of 38 mm, and severe RV systolic dysfunction. There was also tricuspid annulus calcification with a severe tricuspid regurgitation and acceleration time of 89 msec and mean pulmonary artery pressure (PAP) was estimated to be 34 mmHg based on pulmonary valve systolic flow. RV papillary muscles were calcified.

**Figure 1:**
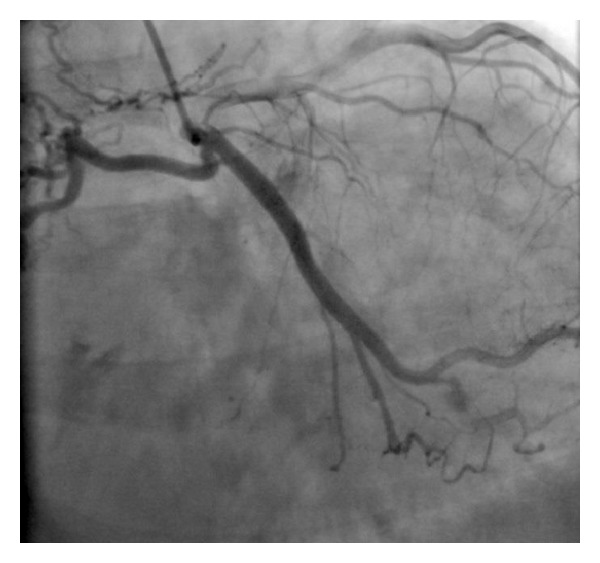
Rich collateral formations between left circumflex artery and right lung assumed as multiple fistulas.

**Figure 2:**
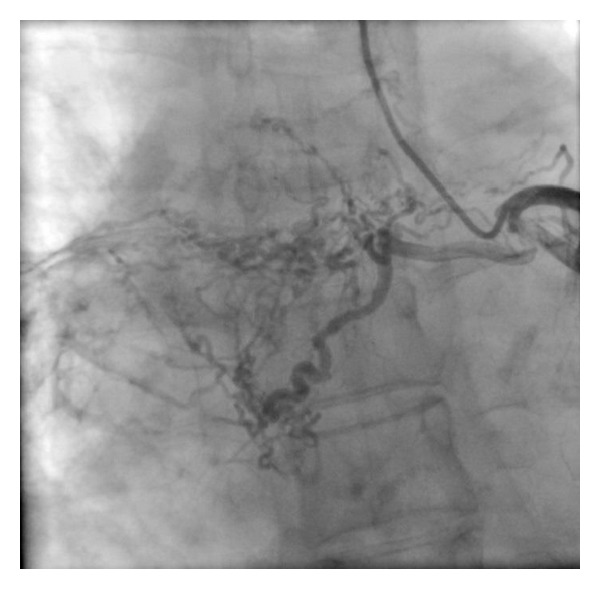
Rich collateral formations between left circumflex artery and right lung assumed as multiple fistulas.

Left heart catheterization and coronary angiography performed via left radial artery revealed normal left and right coronary arteries with rich collateral formations between left circumflex artery and right lung assumed as multiple fistulas (Video 1, Figures 1 and 2; see Supplementary Material available online at http://dx.doi.org/10.1155/2014/427045).

**Figure 3:**
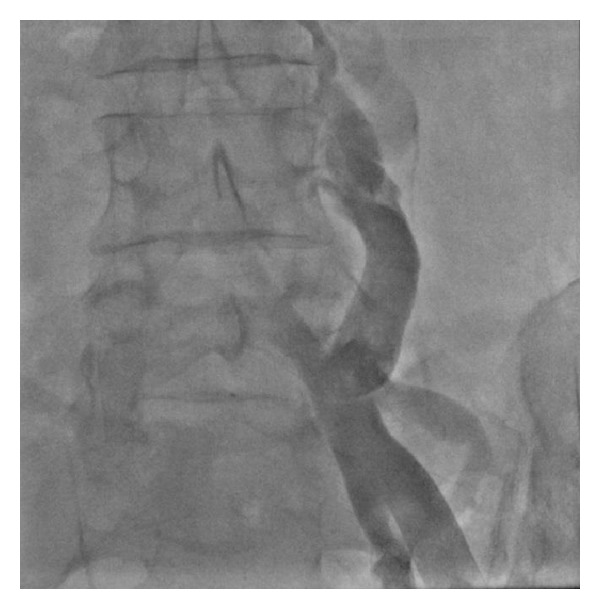
Collateral veins in both right and left sides, draining the lower limb veins through the hemiazygos veins to the right heart.

**Figure 4:**
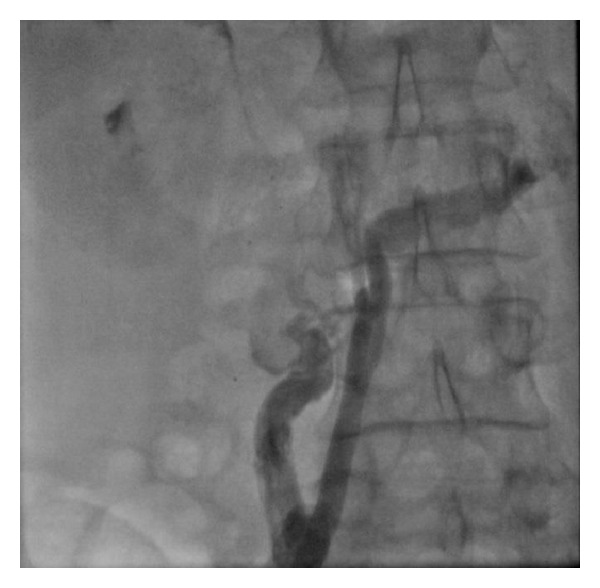
Collateral veins in both right and left sides, draining the lower limb veins through the azygos veins to the right heart.

**Figure 5:**
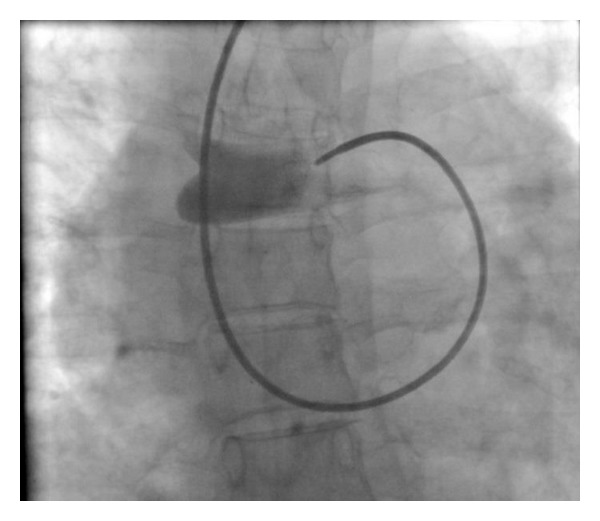
Selective pulmonary angiography revealed proximal occlusion of right pulmonary.

Attempts from both femoral veins for right heart catheterization revealed a cut-off sign at the proximal part of the inferior vena cava (IVC) indicating chronic thrombotic occlusion leading to development of enormous number of collateral veins in both right and left sides, draining the lower limb veins through the hemiazygos (Figure 3) and azygos (Figure 4) veins to the right heart (Video 2). Right heart catheterization was performed via the left subclavian vein using a Swan-Ganz catheter. Selective pulmonary angiography revealed proximal occlusion of right pulmonary artery (Video 3, Figure 5), with RA pressure being 28, RV pressure 42/8–12 mmHg, PAP = 48/20 mmHg, mean PAP = 28 mmHg, PCWP = 20 mmHg, and LV pressure = 140/10–20 mmHg. Flow ratio (*Q*_*P*_/*Q*_*S*_) for calculation of the size of shunt was as follows:

(1) $$\begin{array}{c} \frac{Q_{P}}{Q_{S}}=\frac{\text{Ao}\;{\text{O}}_{2}-\text{MV}\;{\text{O}}_{2}}{\text{PV}\;{\text{O}}_{2}-\;\text{PA}\;{\text{O}}_{2}}, \end{array}$$

where *Q*_*P*_ and *Q*_*S*_ represent blood flows in the pulmonary and systemic circulations, respectively. O_2_ saturations in the right atrium (RA), right ventricle (RV), pulmonary artery (PA), aorta (Ao), and superior and inferior vena cavae (IVC and SVC) were 64%, 65%, 65%, 92%, 62%, and 64%, respectively. Mixes venous (MV) O_2_ saturation was calculated as 62.5% using this formula:

(2) MV O2  saturation =3∗SVC O2  saturation+IVC O2  saturation4.

Because there was not any fistula to the left PA and it was the sole accessible vessel in the pulmonary circulation, the flow ratio was calculated to be about 1.

The ratio of the pulmonary vascular resistance (PVR) to the systemic vascular resistance (SVR) was calculated using this formula:

(3) $$\begin{array}{c} \frac{\text{PVR}}{\text{SVR}}=\frac{\text{PAm}-\text{LAm}}{\text{AOm}-\text{RAm}}\times \frac{Q_{S}}{Q_{P}}, \end{array}$$

where AOm, LAm, Pam, and RAm are the mean pressures of aorta, left atrium, pulmonary artery, and right atrium, respectively. LAm was estimated using pulmonary capillary wedge pressure (PCWP). PVR/SVR was calculated to be 12%.

## 2. Discussion

During attempts to work up a suspected probable pulmonary hypertension, we found rich collateral formations between LCX as well as RCA and right pulmonary artery, primarily assumed as multiple fistulas. Among patients who have chronic thromboembolic pulmonary hypertension, systemic collateral supply to the pulmonary parenchyma has been previously reported to occur from both bronchial and/or nonbronchial systemic circulations [1].

The natural history of coronary artery fistulas is unpredictable. Spontaneous closure secondary to spontaneous thrombosis, although unusual, is a possibility. The management strategy is contentious and most recommendations are based on untrustworthy cases or small retrospective series. Antiplatelet therapy has been advised, particularly in patients with distal coronary artery fistulas and abnormally dilated coronary arteries. Antibiotic prophylaxis against bacterial endocarditis has also been recommended, as bacterial endocarditis is an identified complication. The foremost indications for closure are clinical symptoms of heart failure and myocardial ischemia and sometimes prophylactically in asymptomatic patients with high-flow shunts, to avoid occurrence of symptoms or complications, especially in pediatric population. Surgery and direct epicardial or endocardial ligations were conventionally viewed as the key therapeutic methods for the closure of coronary artery fistulas which is safe and helpful with promising results though treatment of adult asymptomatic patients with nonsignificant shunting is still a matter of debate. Alternatively, catheter-based closure of the fistulous connections as a reasonable nonsurgical option has been recently most popular, with good reported success [2–4].

The adenosine pulmonary reactivity test was not performed because of low pulmonary pressure which had been estimated to be high.

## Supplementary Material

Video 1: Rich collateral formations between left circumflex artery and right lung assumed as multiple fistulas
Video 2: Chronic thrombotic occlusion of the proximal part of the inferior vena cava and collateral veins in both right and left sides, draining the lower limb veins through the azygos and hemiazygos veins to the right heart
Video 3: Proximal occlusion of the right pulmonary artery.
